# Decoding the nanoscale porosity in serpentinites from multidimensional electron microscopy and discrete element modelling

**DOI:** 10.1007/s00410-023-02062-4

**Published:** 2023-10-17

**Authors:** Alireza Chogani, Oliver Plümper

**Affiliations:** Department of Earth Sciences, Utrecht University, Utrecht, The Netherlands

**Keywords:** Serpentinization, Nanogeoscience, Nanoporosity, Confinement, Multidimensional electron microscopy

## Abstract

Serpentinites, widespread in Earth's lithosphere, exhibit inherent nanoporosity that may significantly impact their geochemical behaviour. This study provides a comprehensive investigation into the characteristics, scale dependence, and potential implications of nanoporosity in lizardite-dominated serpentinites. Through a combination of multidimensional imaging techniques and molecular-dynamics-based discrete element modelling, we reveal that serpentinites function as nanoporous media with pore sizes predominantly less than 100 nm. Crystallographic relationships between olivine, serpentine, and nanoporosity are explored, indicating a lack of significant correlations. Instead, stochastic growth and random packing of serpentine grains within mesh cores may result in interconnected porosity. The analysis of pore morphology suggests that the irregular pore shapes align with the crystal form of serpentine minerals. Furthermore, the nanoporosity within brucite-rich layers at the serpentine-olivine interface is attributed to delamination along weak van der Waals planes, while pore formation within larger brucite domains likely results from low-temperature alteration processes. The fractal nature of the pore size distribution and the potential interconnectivity of porosity across different scales further support the presence of a pervasive nanoporous network within serpentinites. Confinement within these nanopores may introduce unique emergent properties, potentially influencing fluid transport, mineral solubility, and chemical reactions. As such, these processes may have profound implications for the geochemical evolution of serpentinites.

## Introduction

Serpentinization of ultramafic rocks has a profound impact on various geochemical and petrophysical processes operating within the oceanic lithosphere of the Earth. For example, serpentinization alters the strength of the lithosphere (e.g., Escartin et al. 2001), seismic properties (e.g., Boudier et al. 2009), geochemical subduction zone input (e.g., Scambelluri et al. 2004), and provides potential energy sources, including hydrogen and methane, for the deep biosphere (e.g., Russell et al. 2010; Schrenk et al. 2013; Nan et al. 2020).

For serpentinization to proceed, fluid pathways must be created in ultramafic rocks, which typically have low permeability. While tectonic deformation and thermal cracking can generate these pathways (Demartin et al. 2004; Rouméjon and Cannat 2014), the mineral reaction itself can also induce differential stress as a result of a significant increase in solid volume (up to 50%), causing the rock to fracture and maintain or increase permeability. This phenomenon has been studied extensively, as demonstrated in the work of, for example, Macdonald and Fyfe (1985), Iyer et al. (2008), Plümper et al. (2012), Kelemen and Hirth (2012) and Malvoisin et al. (2017). While some studies have suggested that serpentinization can occur at a constant volume via mass transport in the fluid away from the hydration site (Fletcher and Merino 2001; Putnis 2009), there is a wealth of geochemical (e.g., near constant Mg/Fe/Si ratios between primary and secondary rock types (Coleman and Keith 1971)), microstructural (Plümper et al. 2012; Malvoisin et al. 2020) and experimental (Klein and Le Roux 2020) evidence indicating a substantial increase in solid volume during serpentinization.

Recent experiments on a serpentinization-analogue system, involving the hydration of periclase to brucite (MgO + H_2_O → Mg(OH)_2_), have demonstrated that under conditions of high reaction rates and low pore-fluid connectivity, hydration reactions may increase permeability by approximately three orders of magnitude (Uno et al. 2022) and reaction-induced stresses could potentially reach gigapascal-levels (Plümper et al. 2022). These findings differ from previous flow-through serpentinization experiments, which showed a decrease in permeability over several orders of magnitude, mainly caused by the clogging of fluid pathways due to mineral precipitation (Godard et al. 2013; Farough et al. 2016). Nonetheless, it remains to be determined whether the hydration reactions will lead to a positive feedback loop between dissolution, precipitation, and renewed fluid pathway generation, or if they will become self-limiting due to growth-induced pore clogging. In either case, it is likely that other fluid transport processes will be necessary to ensure complete serpentinization.

Tutolo et al. (2016) were one of the first to indirectly infer that serpentinites possess intrinsic nanoscale porosity, using small-angle neutron scattering. These findings were recently corroborated by Malvoisin et al. (2021a), who demonstrated that nanoscale porosity exists at the serpentine-olivine interface in a sample obtained from the Oman Drilling Project. However, the origin, distribution, connectivity, scaling behaviour, and relationship to the reaction products of this nanoscale porosity remain largely unknown, although it may play a crucial role during any stage of the serpentinization process. Deciphering the nature of nanoscale porosity in crystalline rocks is crucial for unravelling the mechanisms of fluid and mass transport (Plümper et al. 2017), the principles underlying abiotic methane formation (Le et al. 2017), and the behaviour of geo-fluids in strong geometric confinement (Cole and Striolo 2019). Consequently, Beinlich et al. (2020) have suggested that mass transport through nanoscale porosity could be critical during the carbonation of altered mantle rocks (Kelemen and Matter 2008; Plümper and Matter 2023). The importance of nanoscale porosity for modelling serpentinization has also been addressed in Malvoisin et al. (2021b). Additionally, the porous network that is formed during serpentinization can serve as a conduit for subsequent low-temperature (< 150 °C) alteration processes, which may be vital for producing hydrogen and supporting subsurface microbial activity (Templeton and Ellison 2020). Therefore, it is crucial to differentiate between serpentinization-induced porosity generation and the pore space generated by low-temperature weathering (Jöns et al. 2017; Pujatti et al. 2023).

Here, we employ a combination of multiscale two-dimensional and three-dimensional electron microscopic imaging techniques, along with molecular-dynamics-based discrete element simulations, to provide further insight into the nature of heterogeneous nanoporosity in lizardite-dominated serpentinites. Our findings reveal that nanopores with diameters below 10 nm are prevalent in serpentinite veins, with occasional observation of larger pores measuring up to 100 nm in diameter. Using machine-learning-based image processing techniques and shape descriptors, we quantify the properties of the porous network and its scaling behaviour. Subsequently, we develop a model based on the random packing of granular lizardite crystals to identify the origin and connectivity of pores that may arise during serpentinization.

## Methods

For our study, we selected samples from two different serpentinites locations, which represent in situ oceanic lithospheric mantle and ‘fossil’ exhumed mantle on land. The partially serpentinized peridotite and dunite, respectively, were sourced from (1) the Ocean Drilling Program (ODP) LEG 209 site 1274 (31 km north of the 15^o^20’N Fracture Zone at the Mid-Atlantic Ridge (MAR)) with sample number 209-1274A-11R-1, 56–65 cm (Bach et al. 2006) and (2) the Røragen ultramafic complex, Norway (Plümper et al. 2014). Past examinations using petrography and Raman spectroscopy have determined that the serpentinites from both locations are predominantly composed of lizardite.

Electron backscatter diffraction (EBSD) data were acquired using a Zeiss Gemini 450 scanning electron microscope (SEM) fitted with an Oxford Instruments Nordlys 2 CCD camera. EBSD maps were acquired using an accelerating voltage of 30 kV, probe current of 9.5 nA, and step sizes of 0.2 and 0.4 µm. The orientation data were processed using the Oxford AZtecCrystal software. Unindexed pixels or single pixels were matched by removing different phases from the EBSD dataset and filling unindexed pixels with the average orientation of their grain neighbours. The optimum half-width for contoured EBSD pole figures was determined to be 10°.

To prepare electron-transparent foils for (scanning) transmission electron microscopy ((S)TEM), we used a Thermo Fischer Scientific (TFS) Helios Nanolab G3 focused ion beam scanning electron microscope (FIB-SEM). (S)TEM imaging was performed using both bright-field TEM (BF-TEM) and high-angle annular dark field STEM (HAADF-STEM) modes. For this imaging, we utilized a TFS Talos F200X, a TFS Talos L120C, and a TFS Spectra 300 at acceleration voltages of 200 kV, 120 kV, and 300 kV, respectively. We employed energy-dispersive X-ray (EDX) spectroscopy to determine the elemental composition of the samples.

We also utilized the FIB-SEM to obtain nanotomography volumes using the sequential ion slice and electron imaging technique (e.g., Liu et al. 2016). Electron imaging was conducted in backscattered electron (BSE) mode, with an acceleration voltage of 5 kV and a current of 0.1 nA. For the region1-section1, FIB-SEM nanotomography was performed on a total volume of 17 µm × 12 µm × 4 µm with a voxel size of 3.5 nm × 3.5 nm × 10 nm. For the region1-section3 and region2-section4, the total volumes were 21 µm × 19 µm × 7 µm with a voxel size of 5 nm × 5 nm × 10 nm and 25 µm × 14 µm × 6 µm with a voxel size of 5 nm × 5 nm × 10 nm, respectively. Pore segmentation was accomplished using Ilastik, a machine-learning segmentation toolkit (Berg et al. 2019). Ilastik utilizes a random forest classifier in the learning process, in which a set of nonlinear features characterizes each pixel's neighbourhood. The random forest classifier must be manually trained using user-labelled images as input. This training process is conducted interactively until the segmented images are visually satisfactory and as close as possible to what one manually labels. After segmentation, all FIB-SEM nanotomography volumes were reconstructed and analysed using TFS Avizo. Pore volumes smaller than 50 voxels were excluded from the statistical pore size analyses, while volumes less than 100 voxels were excluded from the shape descriptor analyses. These very small pores often create artificial morphological trends, making it difficult to represent their complex shapes with only a few voxels. Elongation and flatness are measured by dividing the intermediate axis by the longest axis of the best-fit ellipsoid and the shortest axis by the intermediate axis, respectively. These indices are presented in modified Zingg diagrams (Zingg 1935; Angelidakis et al. 2022), where particle classification is overlaid to best characterize pore morphology. In addition, ImageJ/Fiji (Schneider et al. 2012) was employed to perform size and shape analyses for two-dimensional TEM segmented images. Here, pores smaller than 20 pixels and 50 pixels were removed for pore size and shape analyses, respectively.

To avoid filling in nanoporosity with charge-compensating coating materials (e.g., Pt or C), we utilized a Fischione 1061 broad-beam argon ion mill. Imaging was subsequently carried out under low-vacuum conditions (10 Pa). All electron microscopic investigations were conducted at the Utrecht University Electron Microscopy Centre.

For the granular, discrete element simulations, we randomly packed rigid hexagonal lizardite crystals (Mellini 1982) into a 14 × 14 × 50 nm box. Each lizardite crystal had dimensions of 4 × 4 × 2 nm containing 3030 atoms having partial charges based on the ClayFF force field (Cygan et al. 2004). ClayFF is a general force field widely used for molecular simulation of hydrated and multi-component mineral systems and their interactions with aqueous solutions. Interactions between the crystals occurred through contacting spheres, and followed a Hertzian contact law, with tangential friction and collisional energy loss included (Brilliantov et al. 1996). Lizardite crystals were poured into the box with periodic boundary conditions in the x and y directions. The simulation box was bounded using a friction wall boundary condition for the bottom face. Finally, we applied 1 GPa to the system by adding a force in the negative *z* direction to the crystals located at the top part of the box acting like a virtual piston. All granular simulations were conducted using the LAMMPS package (Thompson et al. 2022). For tortuosity analysis of the granular medium, we used TauFactor, a MATLAB application developed by Cooper et al. (2016).

We refer to the term ‘nanoscale porosity’ or ‘nanopore’ when referring to all porosity < 100 nm, following the classification of pore sizes proposed by Mays (2007). This classification is also consistent with other common scientific terminology based on SI prefixes such as nanotechnology (Franks 1987).

## Results

### Crystallographic relationships between olivine and serpentine veins

**Fig. 1:**
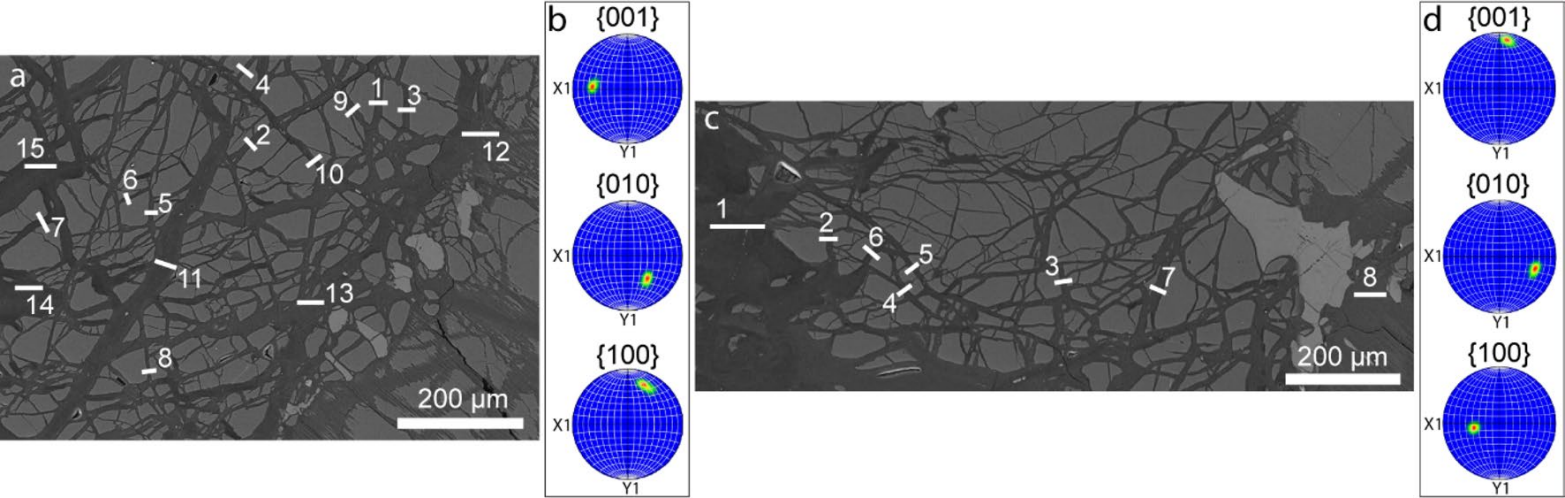
Partially serpentinized peridotite from the MAR (ODP Leg 209). **a** BSE image of region1 (r1) showing locations (1–15) of cross-sections excavated via FIB-SEM. **b** Pole figures depicting the crystallographic orientation of olivine in the r1. **c** BSE image of region2 (r2) showing locations (1–8) of cross-sections excavated via FIB-SEM. **d** Pole figures depicting the crystallographic orientation of olivine in the r2. The dark grey regions in **a** and **c** are serpentine veins, the medium grey fragments are olivine, and pyroxene grains are shown in light grey

**Fig. 2:**
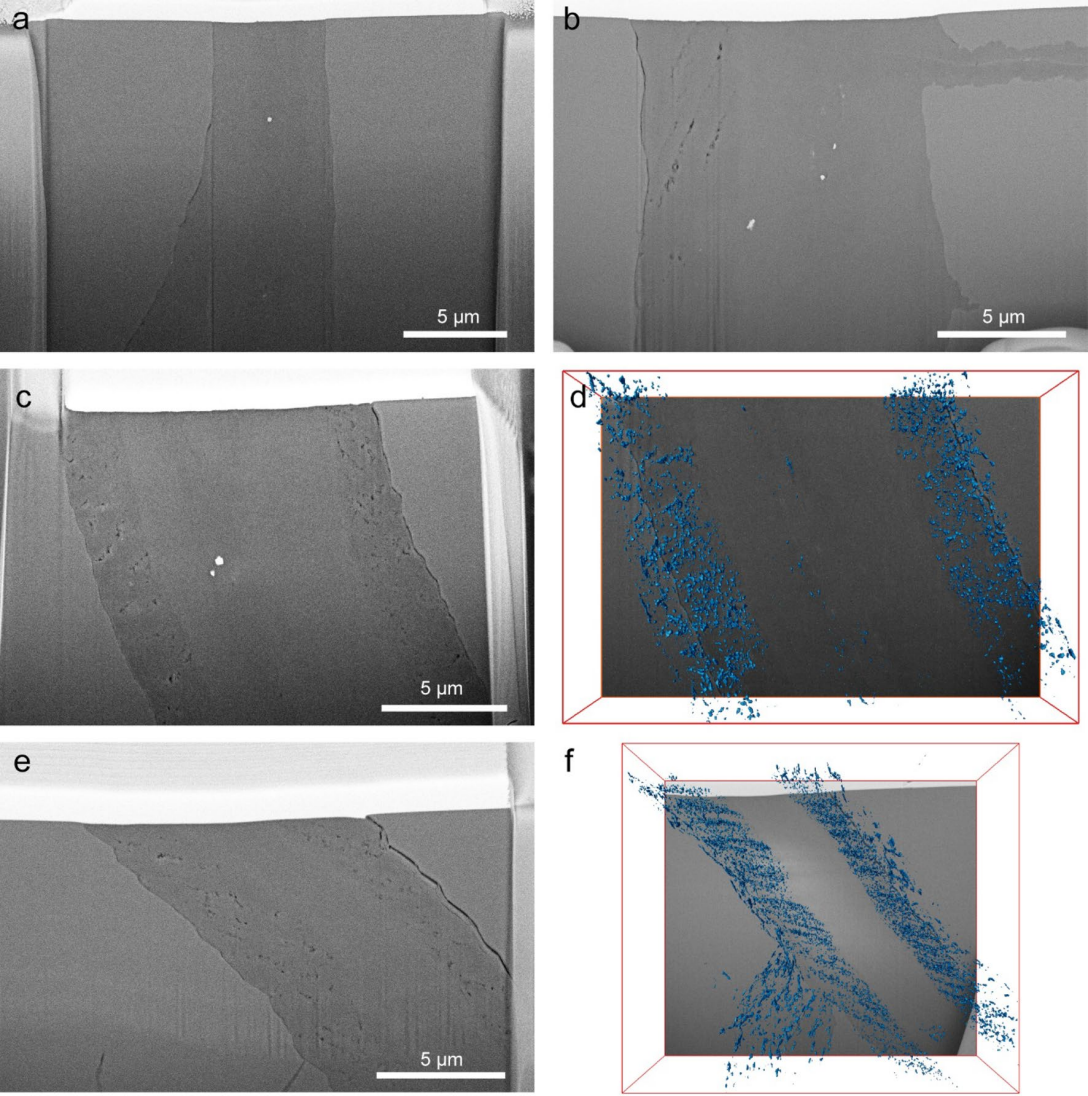
Cross-sectional SEM images and FIB-SEM nanotomography of serpentinite veins. **a** BSE image of an apparently nonporous serpentinite vein cross-section (r1–8). **b** BSE image of a porous serpentinite vein cross-section (r2–4). **c** BSE image of a porous serpentinite vein cross-section (r1–1), pores are in two areas near the reaction front and devoid in the vein centre. **d** Three-dimensional visualization of porosity in r1–1 (total volume: 17 µm × 12 µm × 4 µm), excavated using FIB-SEM nanotomography. **e**, **f** BSE image and three-dimensional visualization of porosity in r1–3 (total volume: 21 µm × 19 µm × 7 µm), respectively, excavated using FIB-SEM nanotomography

To determine the relationships between the overall serpentine vein orientation and the olivine vein walls, we utilized EBSD mapping on samples obtained from ODP Leg 209 (Fig. 1). Based on these EBSD maps, we excavated a total of 23 FIB-SEM cross-sections that are indicated by solid lines in Fig. 1. FIB-SEM cross-sections refers here to the excavation of a ‘trench’ perpendicular to the sample surface to image the excavated face of the sample using electron microscopy. It should be noted that all serpentine vein to olivine orientation relationships are with respect to the vein orientation exhibited in two dimensions on the sample surface. Two regions were investigated, termed region1 (r1) shown in Fig. 1a and region2 (r2) shown in Fig. 1c. Here for r1, sections #1, #3, #5, and #8 were semi-perpendicular to the (001) olivine wall orientation, sections #2, #4, #6, #7, and #11 were semi-perpendicular to the (010) orientation, sections #9 and #10 were semi-perpendicular to the (100) orientation, and sections #12, #13, #14, and #15 were random. In r2, sections #4 and #5 were semi-perpendicular to the (001) orientation, sections #6 and #7 were semi-perpendicular to the (010) orientation, sections #2 and #3 were semi-perpendicular to the (100) orientation, and sections #1 and #8 were random. At an SEM-pixel resolution of ~ 3 nm, most of the 23 cross-sections, we examined appeared to be nonporous. For example, a BSE image of r1 section #8 (r1–8) is shown in Fig. 2a, which reveals no apparent porosity. Only three out of the 23 cross-sections displayed visible pore space (r1–1, r1–3, and r2–4) shown in Fig. 2b–f. BSE images of r1–1 and r1–3 are presented in Fig. 2c, e, where it can be observed that vein porosity is concentrated at the olivine-serpentine interface and absent in the vein centre. In other cross-sections, some pores at interfaces are distinguishable, but the overall serpentine vein appears nonporous. We subsequently conducted FIB-SEM nanotomography of the three porous cross-sections. Three-dimensional visualizations of the pore network in the r1–1 volume and the r1–3 volume are presented in Fig. 2d, f, respectively. The average porosity of the excavated volumes in r1–1, r1–3, and r2–4 were 0.4 ± 0.07%, 0.7 ± 0.08%, and 0.2 ± 0.05%, respectively.

### Nanoscale imaging of porosity in serpentinite vein networks

**Fig. 3:**
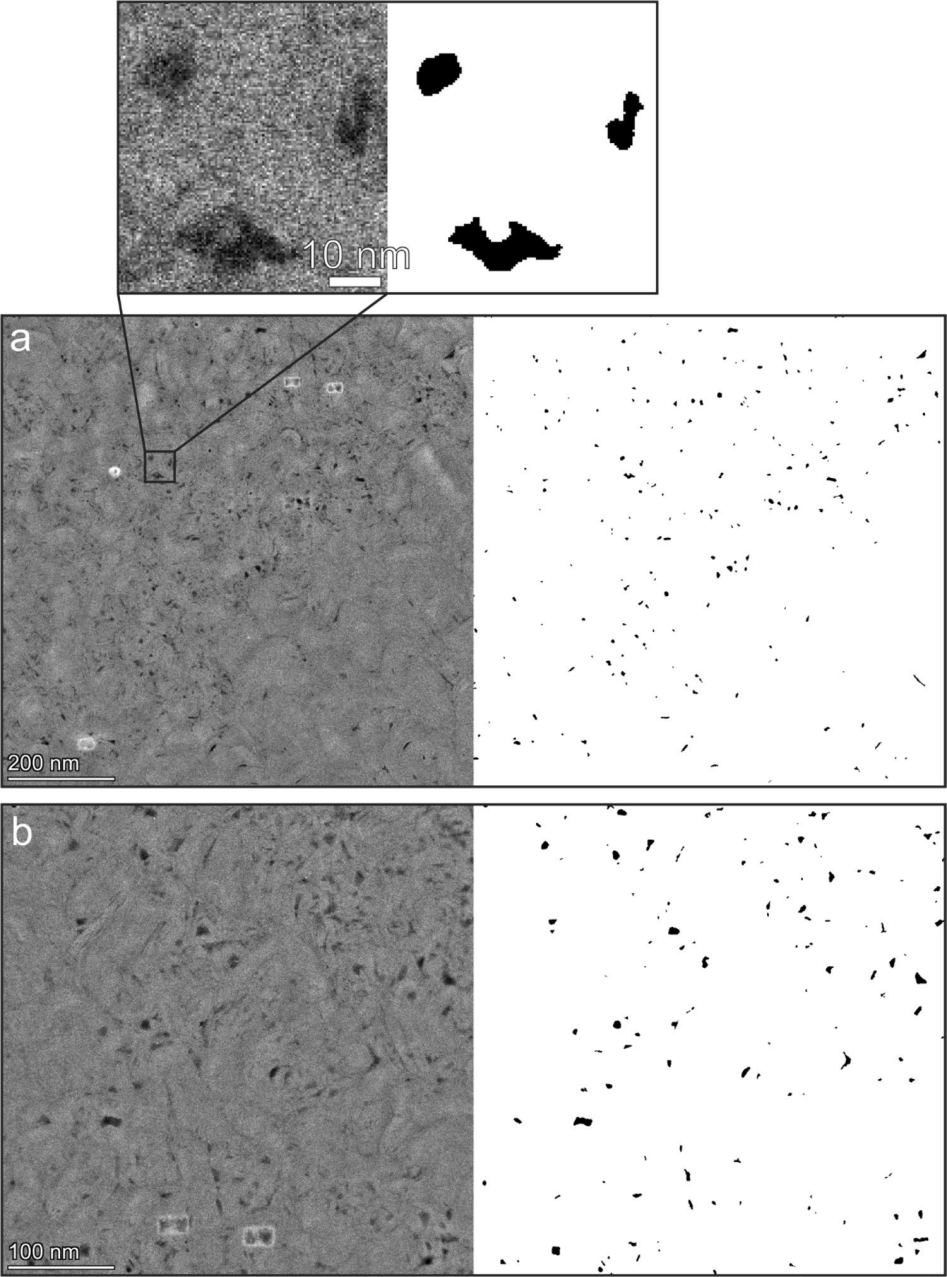
Nanoscale imaging of serpentine porosity and machine-learning-based segmentation. **a**, **b** HAADF-STEM images of region1-section4 foil and their segmented counterparts. Inset depicts the accuracy of the automated, machine-learning-based segmentation approach compared to its real image counterpart

**Fig. 4:**
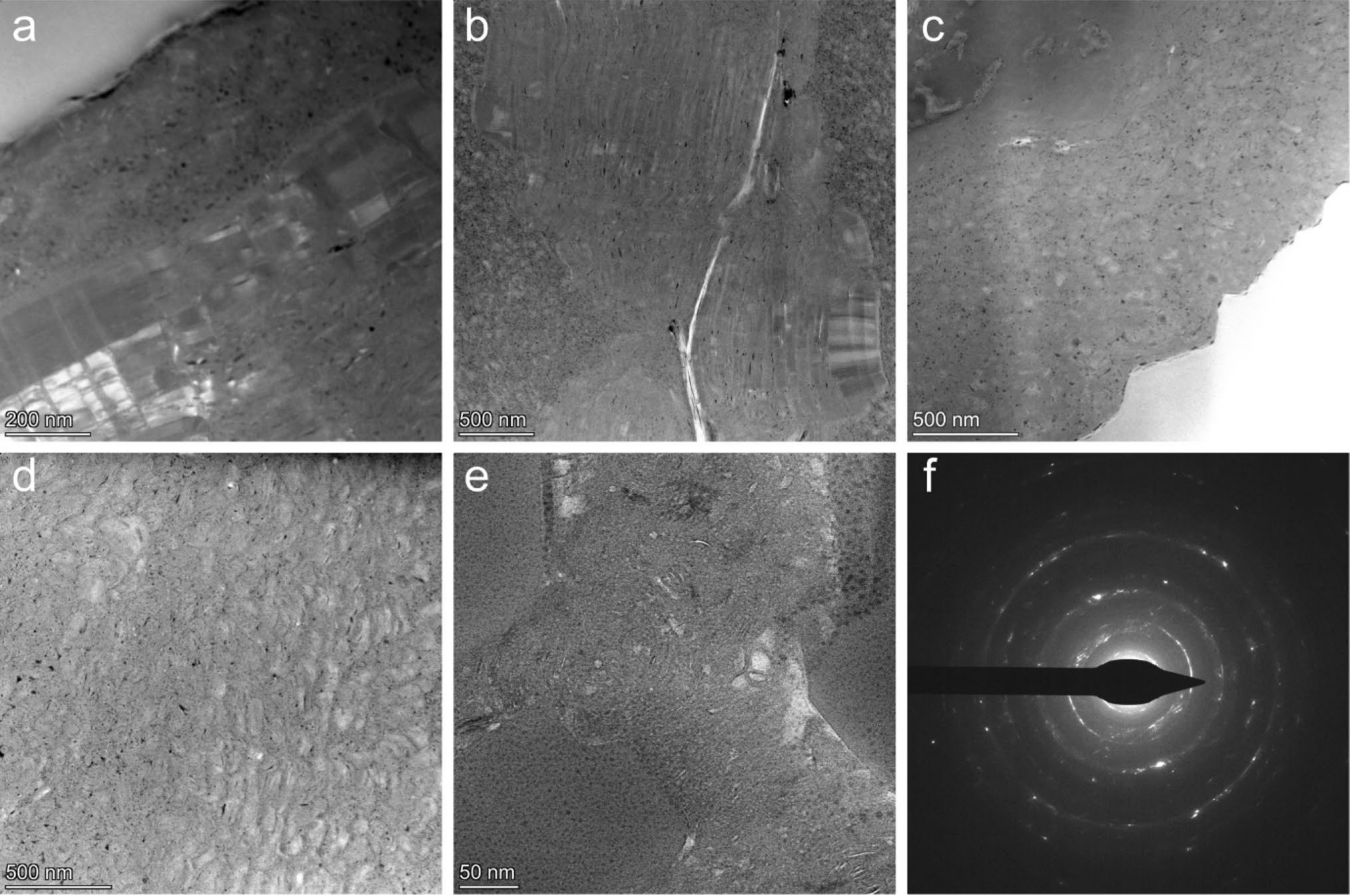
Microstructures of the serpentine veins from TEM imaging.** a**, **b** HAADF-STEM images (sample regions: RL1 and r1–8, respectively) showing a transition from mesh rim columnar lizardite to randomly oriented serpentine mesh core. **c** HAADF-STEM depicting the nanoporous mesh core region adjacent to the olivine-serpentine interface (sample region: r2–6). **d**, **e** Additional microstructures showing the porosity within serpentine veins (sample regions: r1–5 and RL1, respectively). **f** Selected area electron diffraction pattern (sample region: r1–5) highlighting the polycrystalline nature of the mesh cores

To further investigate the porosity in the MAR-serpentinite beyond the resolution of FIB-SEM, we conducted TEM analysis on six electron-transparent foils from different cross-sections of EBSD map region1, one foil from region2, and two foils from locations (RL1 and RL2) within serpentine domains outside of acquired EBSD map regions. HAADF-STEM images of the r1–4 foil, alongside their segmented counterparts, are illustrated in Fig. 3. The black regions observed in these images correspond to pores within the serpentinite. To demonstrate the precision of our segmentation method, a portion of Fig. 3a is enlarged, which shows that the segmented porosity aligns well with the real image counterpart. This reaffirms the accuracy of our machine-learning-based segmentation process. Figure 4a–e shows representative microstructures of those TEM foils also used for pore size extraction. We found that all foils had an average porosity ranging from 1 to 3%. Similarly, to the FIB-SEM tomography results of r1–1 and r1–3, mesh core and mesh rim textures were observed in TEM images as well (Fig. 4a–c). We observed two different domains in the serpentine vein, where the mesh rim in the vein centre exhibited lower porosity, while the mesh cores close to the interface showed higher porosity. Figure 4f shows a representative electron diffraction pattern, confirming that the serpentine within the mesh core is polycrystalline and the grains are near-randomly orientated.

### Porosity within brucite-rich domains

We identified two different brucite-rich domains to which porosity is connected. The first domain is restricted to the serpentine-olivine interface and the second domain occurs throughout the serpentinite. Below we elaborate on their characteristics.

**Fig. 5:**
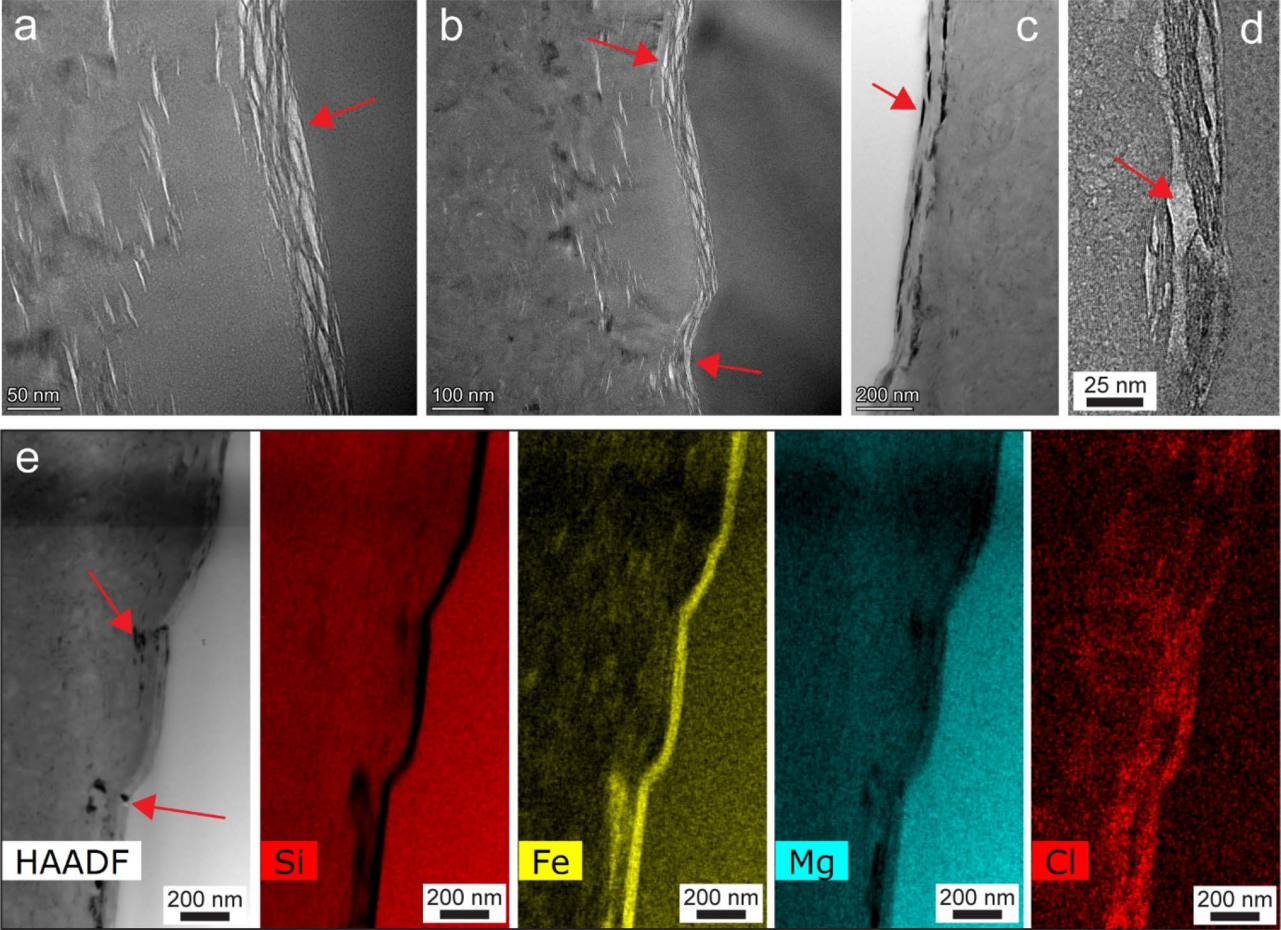
Porosity in brucite layers at the serpentinite-olivine interface (MAR sample).** a**, **b** BF images showing a highly porous layer at the serpentine-olivine interface (sample region: r1–5). **c** HAADF-STEM image of the serpentine-olivine interface (sample region: r1–5). **d** BF image of the interface (sample region: RL1). **e** HAADF-STEM image and EDX elemental maps including Si, Fe, Mg, and Cl showing an iron-rich brucite layer at the interface (sample region: r1–5). Red arrows show porosity

Through EDX-TEM analysis, we discovered a 50–100 nm wide Fe-enriched layer situated at the interface of olivine and serpentine, as depicted in Fig. 5. Analysis of the EDX data suggests the presence of ferroan brucite ([Mg_*x*_,Fe^2+^_1-*x*_]OH_2_) and iowaite (Mg_6_Fe^3+^(OH)_16_Cl_2_∙4H_2_O). This layer is occasionally punctuated with pores, while most layers are riddled with pores primarily oriented sub-parallel to the olivine-serpentine interface. The interface from sample domain r1-5, shown in Fig. 5a–c, displays a pronounced average porosity of 12 ± 4%, representing a tenfold increase compared to mesh core regions. Furthermore, pore sizes often surpass 10 nm. For example, in sample r1–5 interface, 82% of the pores exceed 10 nm in size.

**Fig. 6:**
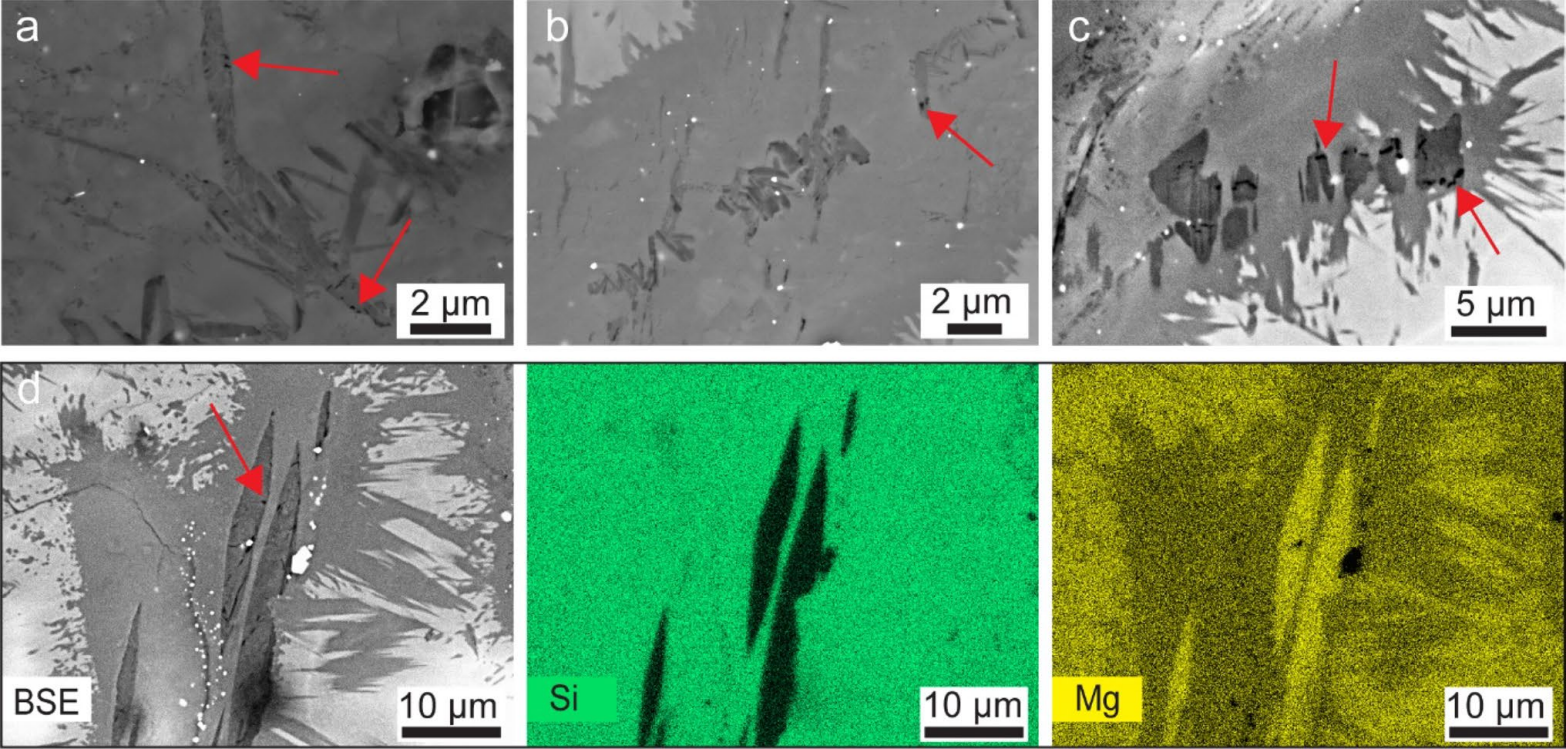
Brucite domains in lizardite-serpentinite (Røragen sample).** a–c** BSE images of uncoated and Argon ion polished serpentinite from Røragen, Norway. Red arrows show porosity in brucite domains. **d** BSE image and EDX elemental maps including Si and Mg showing porous brucite domains

**Fig. 7:**
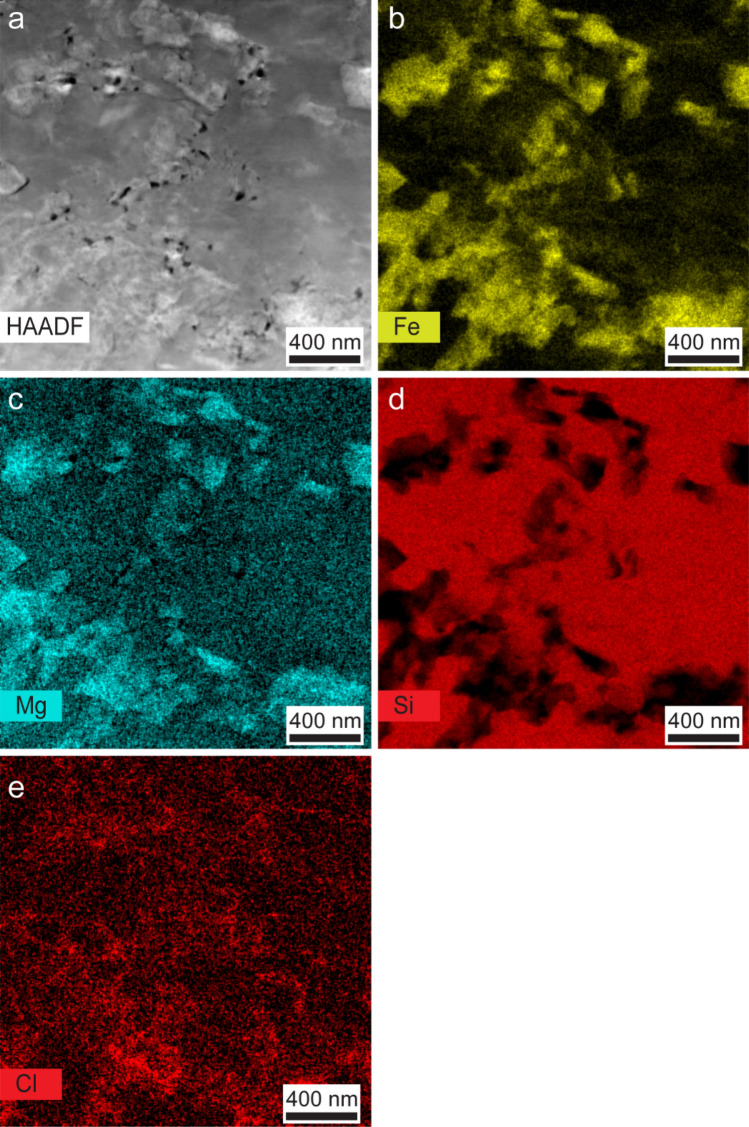
Nanoscale imaging of porosity connected to brucite domains (MAR sample).** a** HAADF-STEM image (sample domain: r1–15) in which the phase with the brighter contrast is brucite and pores are highlighted in black. **b**–**e** Chemical imaging via EDX confirms that the bright areas in **a** are enriched in Mg and Fe and depleted in Si indicating the presence of brucite

Contrary to the interface brucite layer, we also identified elongated, blade-like brucite domains within the bulk serpentinite (Fig. 6). Here, we specifically focused on ion-beam polished samples as the polished revealed elongated brucite domains, previously not identified using mechanically polished sample preparation. SEM imaging of the ion-beam polished surfaces revealed an elevated porosity distribution within the brucite-bearing domains, preferentially located at the brucite-serpentine interface. Subsequent, nanoscale imaging with the TEM (Fig. 7) confirmed the presence of nanoporosity within these brucite domains. Also, here the porosity appears to be predominately situated at the interface between brucite and serpentine.

## Discussion

Our findings substantiate that lizardite-serpentinites inherently exhibit nanoporosity. In fact, nanopores are omnipresent and play an essential role in the overall porosity of many geological materials (e.g., Anovitz et al. 2009, 2013; Wang 2014; Plümper et al. 2017). While both indirect and direct analytical methodologies have been employed to examine nanoporosity in serpentinites, a comprehensive investigation into their characteristics, size distribution, and scale dependence remains largely unexplored. Furthermore, there appears to be an absence of a robust model elucidating the genesis of nanoporosity in serpentinites, particularly in lizardite-dominated serpentinites.

### Crystallographic relationships between olivine, serpentine and nanoporosity

A plethora of research has shown that the alteration, and as such serpentinization, of olivine adheres to an interface-coupled dissolution–precipitation mechanism (e.g., King et al. 2010; Plümper et al. 2012), involving the dissolution of olivine and precipitation of serpentine, often through metastable precursor phases (e.g., Rumori et al. 2004; Lafay et al. 2016). This process is widely recognized to occur during fluid-driven mineral replacement reactions (Putnis 2009). Based on the fluid chemistry, it is possible to uncouple the dissolution and precipitation processes from the reacting interface (Xia et al. 2009). The generation of porosity appears to be an intrinsic aspect of fluid-driven replacement, typically attributed to a negative molar volume change or a difference in solubility (e.g., Putnis 2009). In a system exhibiting epitaxy between the reactant and product, this porosity adheres to specific crystallographic relationships. For instance, in feldspar replacement reactions, the inherent nanoporosity exhibits strong anisotropy and aligns with the [100] direction (Plümper et al. 2017).

During the serpentinization process, considerable positive molar volume changes can result in reaction-induced fracturing (e.g., Plümper et al. 2012), wherein subsequent crystal growth is anticipated to occupy any pre-existing or newly created pore space. Nevertheless, despite the prediction that crystal growth would ultimately fill any pore space, our study, in corroboration with others (e.g., Tutolo et al. 2016; Malvoisin et al. 2021a), reveals that lizardite-serpentinites inherently exhibit porosity. To disentangle a potential role of crystallographic relationships between lizardite/chrysotile and olivine on the nanoporosity one needs to review the generation of the omnipresent mesh texture within lizardite-dominated serpentinites. The mesh texture (Fig. 1a, c) has been interpreted to arise from various stages of hydration, changing conditions during olivine serpentinization, or recrystallization of poorly crystalline hydration products (Dungan 1977; Wicks et al. 1977; Cressey 1979; Viti and Mellini 1998). Overall, a two-step process results in the formation of a mesh rim and mesh core. Here, the mesh rim is identified by columnar lizardite grains that grow preferentially with their [001] axis normal to the olivine interface. Although, there is a dominant preferred orientation of the lizardite grains, numerous electron microscopic studies show that the olivine-to-lizardite relationship is non-topotactic (e.g., Rumori et al. 2004; Boudier et al. 2009). It is the grain boundaries between the columnar lizardite grains that may allow fluid to diffuse to the reaction interface as outlined in Malvoisin et al. (2021a). However, mesh rims are typically not broader than a few micrometres and a transition to mesh cores results in unoriented, random growth of primarily lizardite and chrysotile as shown in Fig. 4. As discussed in Viti and Mellini (1998) the transition from mesh rim to the core may correspond to an overall evolution of the serpentinization system’s permeability and hence the availability of fluid at the reaction interface. As such, the switch in rim-to-core serpentine growth could result from a change in olivine dissolution kinetics not being the rate-limiting step but secondary serpentine precipitation, similar to other mineral replacement reactions (e.g., Xia et al. 2009). Nonetheless, we explored the potential impact of olivine crystallography, which is defined by a higher dissolution rate along the [010]-axis in comparison to the [001]- and [100]-axes (Awad et al. 2000). We also considered the preferential formation of dissolution structures like etch pits, roughly parallel to (100), (010), and (001) surfaces (Kirby and Wegner 1978). These factors were examined in relation to their possible effects on the inherent nanoporosity characteristics, especially within the partially formed mesh cores found in the serpentine vein network.

**Fig. 8:**
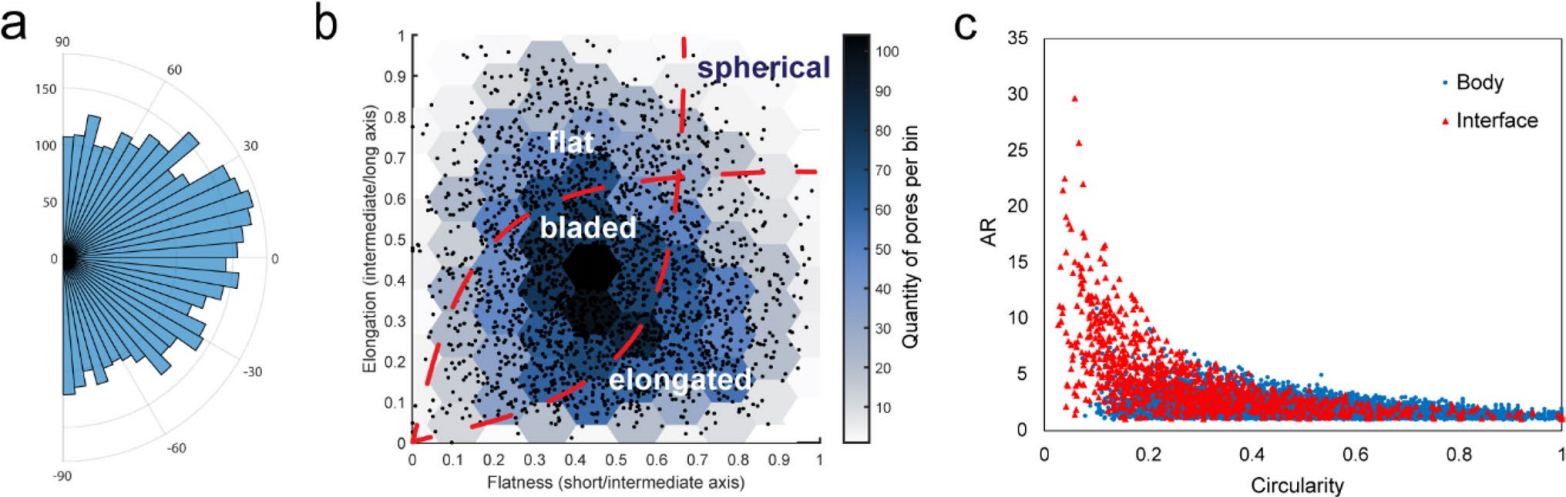
Pore shape and orientation analysis of serpentinite porosity.** a** Polar histogram showing the orientation of mesh-core porosity from TEM images. In this plot, 0 degree represents pores parallel to the serpentine-olivine interface and ± 90 represents pores perpendicular to the interface. **b** Modified Zingg diagram of pores from FIB-SEM tomography (sample region: r2–4). **c** Shape comparison of body and brucite-layer interface pores (sample region: r1–5)

Comparing olivine interface orientation, pore sizes and shape descriptors do not reveal any obvious correlations. Comparison of pore orientations within the mesh cores (Fig. 8a) with respect to the serpentine-olivine interface from two-dimensional TEM images does not show any preferred alignment with respect to the reaction interface. The pore orientation distribution appears uniform, suggesting that the porosity stems from a random growth of crystals, confirmed by electron diffraction (Fig. 4f). Overall, our analysis of orientation relationships between olivine, serpentine and nanoporosity did not reveal any significant correlation that could be associated with dissolution rates or dissolution structures of the reactant.

### Tracing the development of mesh-core serpentine porosity via pore morphology indicators

The above information indicates that there seems to be no discernible relationship between the porosity in the serpentine vein network and the crystallographic connections between reactant and product. Yet, the shape and structure of the pores could provide key insights into their origin.

To gain a comprehension of the pore shapes within the FIB-SEM nanotomography volumes, we utilized a modified Zingg diagram (Fig. 8b; Zingg 1935; Angelidakis et al. 2022). Here, elongation is determined by dividing the intermediate axis by the longest axis of the ellipsoid that best fits the pore, while flatness is calculated by dividing the shortest axis by the intermediate axis. As evident from the Zingg diagram, most pores exhibit a bladed shape.

As we cannot apply the same approach to two-dimensional data extracted from the TEM images (Figs. 3, 4), we employed the following shape descriptors (Table 1); aspect ratio (AR), circularity, and solidity. Here, AR is defined as the ratio of the major axis to the minor axis of the pore’s fitted ellipse. Circularity measures how closely the shape of a pore approaches that of a mathematically perfect circle and solidity measures the overall concavity of a pore by dividing the total pore area by its convex area. The average aspect ratio and circularity of the pores in the TEM foils were 2.15 ± 0.32 and 0.51 ± 0.09, respectively. These values suggest that the serpentine pores are generally elongated rather than circular. Circularity values indicate that the pores are not perfectly circular but are somewhat irregular in shape. In comparison, porosity within the brucite-rich layers at the serpentine-olivine interface exhibits consistently higher AR values and lower circularity (Fig. 8c).

**Table 1:** Shape descriptors of serpentine pores imaged using the transmission electron microscope

TEM Foil	Porosity %	Circularity	AR	Solidity	Average Diameter (nm)
r1–4	0.9 ± 0.3	0.64 ± 0.17	2.05 ± 0.95	0.84 ± 0.08	4.6 ± 2.3
r1–5	2.3 ± 1.9	0.47 ± 0.18	1.96 ± 0.85	0.77 ± 0.1	4.7 ± 3.6
r1–7	1.0 ± 1.0	0.35 ± 0.18	2.93 ± 2.66	0.70 ± 0.10	10.8 ± 11.1
r1–8	2.3 ± 1.6	0.59 ± 0.19	2.01 ± 1.07	0.82 ± 0.19	6.5 ± 5.3
r1–9	3.5 ± 2.1	0.42 ± 0.20	1.97 ± 0.99	0.72 ± 0.11	3.2 ± 2.2
r1–15	1.0 ± 0.1	0.46 ± 0.18	2.36 ± 1.21	0.78 ± 0.11	16.7 ± 13.2
r2–6	2.9 ± 1.0	0.60 ± 0.20	1.85 ± 0.91	0.83 ± 0.19	6.3 ± 4.3
RL1	2.3 ± 0.8	0.48 ± 0.21	2.28 ± 1.43	0.77 ± 0.12	4.9 ± 4.8
RL2	1.4 ± 0.7	0.56 ± 0.19	1.94 ± 0.83	0.80 ± 0.09	3.5 ± 2.8

Overall, the analysis of pore morphology suggests that the irregular shapes of the pores within the serpentine mesh cores may be attributed to the underlying mineral formation process. Hence, this suggests that the creation of the observed porosity may not be through processes such as dissolution or cracking, which typically result in elongated channel-like pore structures (Hoefner and Fogler 1988; Yang et al. 2020; Wang et al. 2022; Pujatti et al. 2023), but is more likely directly related to the inherent crystal form of the serpentine minerals themselves.

### Porosity within the brucite layer at the interface and within brucite domains

Our study reveals two distinct types of pore structures associated with brucite. The first type is linked to the interface region between serpentine and olivine. This observation is consistent with what Klein et al. (2009) observed when they detected brucite domains adjacent to olivine fragments in a MAR sample from the same site investigated here. Their thermodynamic analyses suggest that as the water-to-rock ratio decreases, brucite tends to have a higher Fe content. This may account for our observed Fe-enriched brucite layer at the boundary, which may developed before the reaction came to a halt. Through TEM images and morphological descriptors, the pore space appears to align sub-parallel to the basal plane of brucite. This suggests that it is likely a result of delamination along the weakest van der Waals planes within the brucite crystal. Such delamination is probably due to pressure relief during the progressive 'unloading' of the serpentinite as it approaches or reaches the Earth's surface. Furthermore, the intermittent drying of the sample either within the lithosphere or during sample preparation could have resulted in similar delamination porosity, mirroring the phenomena observed in clay (Desbois et al. 2009; Rodriguez et al. 2014; Yu et al. 2021). In general, decompression leads to fractures and affects macroscopic-scale properties of rocks, such as the elastic modulus, without significantly altering their microscopic structures (Auzende et al. 2015; Wei et al. 2017). The second type of porosity is primarily observed within larger brucite domains and presents a near-circular morphology. It is located at the brucite–serpentine interface. We propose that this type of porosity is likely due to the preferential dissolution of brucite at low temperatures, resulting from weathering processes either near the ocean floor or on the Earth's surface. This hypothesis is supported by Jöns et al. (2017) and recent findings from Klein et al. (2020), who demonstrated that brucite dissolution frequently impacts large portions of serpentinites exposed to seawater. Alternatively, the replacement of brucite by iowaite, which is inferred from the increased levels of Cl (Fig. 5), might have contributed to the creation of porosity.

### Scaling behaviour of the serpentinite porosity from multidimensional imaging

**Fig. 9:**
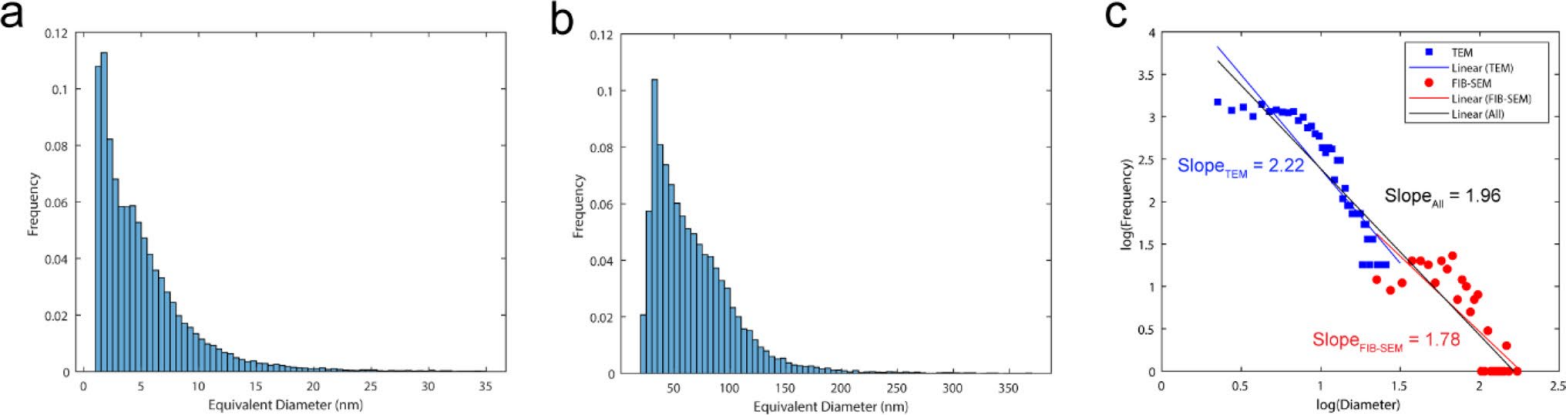
Pores size and fractal analysis** a**, **b** pore size distributions extracted from TEM images and FIB-SEM nanotomography volumes, respectively. **c** Multiscale fractal analysis incorporated both TEM and FIB-SEM nanotomography data

Finally, we examine the distribution of pore sizes across all specimens analysed via three-dimensional FIB-SEM nanotomography and two-dimensional TEM imaging. Statistical analysis of the porosity (Fig. 9a, b) reveals that the dominant pore diameter is < 100 nm.

Fractal geometry concepts have been extensively employed to characterize and quantify irregularities (Mandelbrot 1982; Turcotte 1989), specifically in the size distribution of grains and pores observed in natural phenomena (e.g., Houben et al. 2013; Ohl et al. 2021). We utilize the slope of the log(frequency)-log(diameter) histogram to assess the fractal properties of serpentinite porosity. Our analysis demonstrates that the pore size distribution exhibits fractal characteristics, with an average fractal dimension *D* = 2.17 ± 0.56 for all TEM analyses and a *D* = 2.29 ± 0.19 for FIB-SEM nanotomography volumes. These findings suggest that the observed serpentinite porosity at various scales can be attributed to the same underlying processes.

To further investigate the scaling behaviour of porosity, a FIB-SEM image and a corresponding TEM image were integrated (sample domains: r1–1 and r1–5 for FIB-SEM and TEM images, respectively), and the frequency was computed with 20 bins per order of magnitude of pore size. The bin widths for the TEM and FIB-SEM scales were set at 0.5 nm and 5 nm, respectively. The porous region in the SEM image covered an area of 54 µm^2^, while the area of the TEM image spanned 3 µm^2^. Consequently, the frequency of the TEM image bins was multiplied by 18. The outcomes from FIB-SEM nanotomography and TEM imaging were amalgamated into a single diagram (Fig. 9c), which demonstrates a strong correlation between the two scales. This further supports the fractal nature of the pore system and the congruity in the pore formation mechanism at varying scales.

### Nanoscale porosity in lizardite-dominated serpentinites: a model

Thermomechanical and reaction-induced cracking have been widely acknowledged as critical mechanisms in initiating fluid pathways during serpentinization. However, numerous studies emphasize the significance of nanoporosity in serpentinites, although a comprehensive model describing porosity generation remains elusive. Malvoisin et al. (2021a) investigated the exploration of fluid pathways in serpentine porosity, positing that grain boundaries between columnar lizardite grains in mesh rims are instrumental in facilitating fluid diffusion to the olivine reaction interface. While oriented growth and subsequent directional diffusion are vital for serpentinization progression, most of the porosity in serpentinites, beyond the initial stage of mesh rim growth, is observed within randomly developed lizardite/chrysotile mesh cores. We propose that this stochastic growth and its intrinsic porosity play a pivotal role in driving the serpentinization process towards completion.

To assess the potential interconnectivity of porosity within mesh cores and the subsequent facilitation of fluid movement through a nanopore network, acquiring three-dimensional data sets is crucial. However, the inherent beam sensitivity of serpentine and its nanoscale characteristics preclude the collection of such data, especially using transmission electron microscopy-based nanotomography. Consequently, we employ numerical simulations to investigate the possible connectivity of the nanoporous mesh core.

**Fig. 10:**
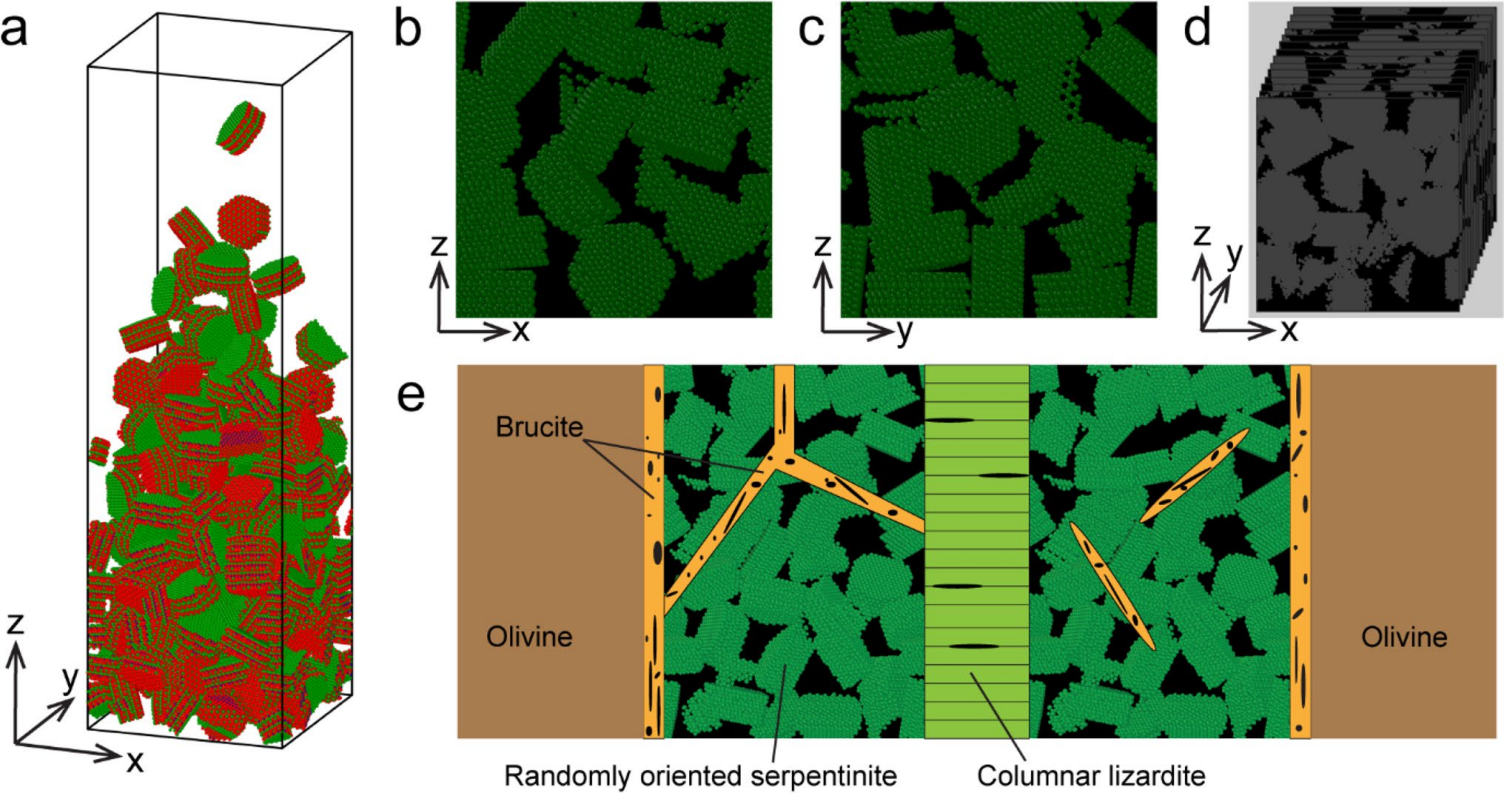
Model for nanoporosity in serpentinite mesh cores. **a** Molecular-dynamics-based granular model with lizardite crystals randomly filling the model box. **b**, **c** Orthogonal slices showing porosity (dyed black) between lizardite crystals (dyed green). **d** Binary segmented reconstructed volume (pores and solid dyed black and grey, respectively). **e** Schematic model of porosity in serpentinite. Potential pathways for fluid flow toward the reaction front are (1) nanoporosity in serpentinite mesh cores (2) grain boundaries between columnar lizardite (3) porous brucite-rich domains

Our simulation is designed to understand how a random assembly of grains may form a pore network, even under confining pressure. We suggest that the random growth patterns of lizardite and chrysotile, corroborated by electron diffraction (Fig. 4f), can be paralleled by simulating granular material stacking in confined conditions. Such random configurations align with observed pore shapes that echo crystal faces, evident from the bladed, low-circularity pore morphology (Fig. 8). While this methodology simplifies the intricate fluid-driven mineral replacement process, it provides foundational insights into nanoscale porosity within the serpentinite mesh core. Our model prioritizes the end pore structure rather than the actual growth dynamics. We postulate that during reactions, serpentine grains nucleate heterogeneously and grow in varied directions. This haphazard growth, marked by imperfect grain alignment, culminates in intergranular porosity. This phenomenon is not exclusive; similar patterns emerge in numerous other fluid-driven mineral replacements, where random three-dimensional heterogeneous nucleation overshadows topotaxial growth (e.g., Cubillas et al. 2005). We observe no recrystallization, which would otherwise diminish porosity and align serpentine grains. Consequently, random growth can be likened to the progressive packing of grains in limited space (Fig. 10a). Hence, we employed a molecular dynamics-inspired approach with granular medium simulations (Fig. 10). Here, lizardite crystals are placed into a rectangular confined space. Each crystal consists of individual atoms with partial charges. To investigate the potential effect of confining pressure on pore closure, we applied a 1-GPa uniaxial load to the granular medium. Figure 10b, c shows two orthogonal slices in the middle of randomly packed lizardite crystals volume under 1 GPa uniaxial load. Lizardite crystals are green, and pores are represented in black. Figure 10d shows a reconstructed binary segmented volume of randomly packed serpentinite crystals (pores and solids dyed in black and grey, respectively).

Tortuosity, an intrinsic property of a porous medium defined as the ratio of the actual flow path length to the straight-line distance between the endpoints of the flow path, can help us comprehend the potential structure and connectivity of the pore network within the simulated volume of lizardite-serpentinite. We utilized the tortuosity factor τ, a dimensionless measure that describes the impact of the solid phase's morphology on fluid flow. The values for tortuosity can be determined along any direction, and in the event of a completely disconnected pore network, it tends towards infinity in that direction (Cooper et al. 2016). In the *x*-, *y*-, and *z*-directions, the tortuosity for the simulated case is 3.13, 3.10, and 2.64, respectively. This suggests that in our model, porosity is interconnected in all directions.

While our model suggests high interconnectedness, it is important to consider that the porosity in a natural setting may not be as interconnected. Yet, our discrete element model demonstrates that random packing, serving as an analogue to random growth, is likely to yield substantially interconnected porosity. Future models of porosity development during serpentinization, other than reaction-induced fracturing, should further include the growth dynamics inherent to fluid-driven replacement reactions, the progression of fluid pressure, and the subsequent dynamic changes in porosity and its effects on permeability.

In summary, our combined microstructural observations and numerical simulations suggest that in the absence of reaction-induced fracturing, fluid transport would occur through a variety of pore spaces with diverse characteristics. These range from grain boundary pathways within the columnar lizardite found in the mesh rims (Malvoisin et al. 2021a), to the random pore space present within the mesh cores (Fig. 10e). Future investigations will require advanced techniques like X-ray ptychography (e.g., Holler et al. 2014) to fully determine the extent of interconnectedness between the porosities in the different microstructural domains and across all pore sizes. However, our research supports the notion that most of the porosity in serpentinite is at the nanoscale. Therefore, the confinement of fluids to these tiny spaces may present physical properties that differ from those observed at larger scales.

## Lizardite-serpentinites as nanoporous media: some potential consequences

Our research underscores that serpentinites inherently function as nanoporous media, with pore sizes typically far less than 100 nm. Fluids restricted to these minuscule scales exhibit distinct behaviours different from their bulk counterparts (e.g., Chen et al. 2014; Zhong et al. 2020; Sun et al. 2020). This could have a considerable effect on the geochemical nature of these fluids. Nanoconfinement may also influence fluid and mass transport through electrokinetic effects (e.g., Plümper et al. 2017) and may change the physical properties of the fluid itself. For example, Fumagalli et al. (2018) showed experimentally that the dielectric constant of water drops drastically in nanoconfinement having potentially a first-order effect on mineral solubility, ion transport and chemical reactions. Moreover, reactive ensemble Monte Carlo (Le et al. 2017) and ab initio molecular dynamics (Stolte et al. 2022) simulations suggest that the reduction of carbon dioxide to methane is favoured under confinement and that carbon dioxide and water confined in nanopores may enhance mineral carbonation processes. Hence, confinement effects in serpentinites may have a drastic effect on the geochemical evolution of the serpentinizing system and, e.g., the production of abiotic organics.

## Conclusion

Our comprehensive investigation of nanoporosity in lizardite-dominated serpentinites has provided valuable insights into their microstructural characteristics, crystallographic relationships, and potential implications. The presence of nanopores, ranging from sub-10 nm to 100 nm in size, is a ubiquitous feature in serpentinites and may play a fundamental role in their geochemical behaviour. Crystallographic orientations of olivine, serpentine, and nanoporosity did not reveal significant correlations, indicating that the porosity generation process is complex and not solely governed by crystallographic relationships as observed in other fluid-driven mineral replacement processes.

The analysis of pore morphology, both in the mesh cores and brucite-rich layers, shed light on their origin. Pores within mesh cores exhibited irregular shapes aligned with the crystal form of serpentine minerals, suggesting that they are a direct result of stochastic growth and random packing of lizardite (and chrysotile) grains. On the other hand, pores within brucite-rich layers are likely linked to delamination along weak van der Waals planes and low-temperature alteration processes.

The fractal nature of the pore size distribution observed and the potential interconnectivity from numerical simulations suggest that lizardite-dominated serpentinites may be characterized by a pervasive nanoporous network. The confined fluid behaviour within these nanopores challenges existing geochemical models, highlighting the need for a nanoscale perspective to accurately capture the geochemical dynamics of fluids and their interactions with minerals. Nanoscale confinement may influence fluid transport, mineral solubility, ion transport, chemical reactions, and even the production of abiotic organics, emphasizing the far-reaching implications of nanoporosity in serpentinites.

Future research should focus on unravelling the interconnectedness and spatial distribution of nanoporosity within serpentinites, employing advanced imaging techniques to fully characterize the pore network. Additionally, deciphering size-dependent processes during fluid-rock interaction may significantly advance our knowledge of the geochemical evolution during serpentinization.

## Data Availability

All data are available in a data publication that can be accessed through the Utrecht University YODA portal: https://public.yoda.uu.nl/geo/UU01/ZGEYQY.html.

## Code availability

All information regarding the codes used in this publication is available in a data publication that can be accessed through the Utrecht University YODA portal: https://public.yoda.uu.nl/geo/UU01/ZGEYQY.html.
